# The effect of travel restrictions on the spread of a moderately contagious disease

**DOI:** 10.1186/1741-7015-4-32

**Published:** 2006-12-14

**Authors:** Martin Camitz, Fredrik Liljeros

**Affiliations:** 1 Swedish Institute for Infectious Disease Control, Solna, Sweden; 2 Department of Medical Epidemiology and Biostatistics, Karolinska Institute, Solna, Sweden; 3 Department of Sociology, Stockholm University, Stockholm, Sweden; 4 Theoretical Biological Physics, Department of Physics, Royal Institute of Technology, Stockholm, Sweden

## Abstract

**Background:**

Much research in epidemiology has been focused on evaluating conventional methods of control strategies in the event of an epidemic or pandemic. Travel restrictions are often suggested as an efficient way to reduce the spread of a contagious disease that threatens public health, but few papers have studied in depth the effects of travel restrictions. In this study, we investigated what effect different levels of travel restrictions might have on the speed and geographical spread of an outbreak of a disease similar to severe acute respiratory syndrome (SARS).

**Methods:**

We used a stochastic simulation model incorporating survey data of travel patterns between municipalities in Sweden collected over 3 years. We tested scenarios of travel restrictions in which travel over distances >50 km and 20 km would be banned, taking into account different levels of compliance.

**Results:**

We found that a ban on journeys >50 km would drastically reduce the speed and geographical spread of outbreaks, even when compliance is < 100%. The result was found to be robust for different rates of intermunicipality transmission intensities.

**Conclusion:**

This study supports travel restrictions as an effective way to mitigate the effect of a future disease outbreak.

## Background

Knowledge of the speed at which a contagious disease travels between geographical regions is vital for making decisions about the most effective intervention strategies. The actual routes a disease will take are strongly determined by how individuals travel within and between regions [1-4]. As was shown during the outbreak of severe acute respiratory syndrome (SARS) [5], current travel patterns enable contagious diseases to spread to far corners of the globe at alarming rates. This demonstrates the need for a new type of model that incorporates travel networks.

Several authors have responded to the call, resulting in now-classic papers. Rvachev and Longini [6], with their followers Grais et al [7], were among the first to publish such studies, using deterministic models. Hufnagel et al [8] have demonstrated how a simple stochastic model in conjunction with data on aviation traffic could be used to simulate the global spread of the SARS epidemic. Using a stochastic transmission model on both a city level and globally, with each city interconnected by the international aviation network, they produced results in surprising agreement with World Health Organization (WHO) reports of the actual epidemic. With some exceptions, most prominently Japan, all infected countries in the simulation were also present in the WHO reports. The orders of magnitude were also closely matched.

Our study applied a version of the Hufnagel model to Sweden in order to predict the effect that travel restrictions might have on the geographical spread of an outbreak. Instead of using only the aviation network, which connects only some 30 towns in Sweden, we used survey data on all intermunicipal travel, including all forms of travel.

Sweden is, by European standards, a large country, with a small population. Just over 9 million people share 450000 square kilometers. The population is, however, largely urbanized, and in that respect similar to other industrialized nations with large areas.

Eubank et al [9] estimated a travel network at community level using census data. Our data directly cover traveling over the whole nation on all scales, although we have kept only the regional data. It is sufficiently extensive that simulation or smoothing for estimating a travel network could be avoided and thus can, in some respects, be regarded as "real". Using such data in a simulation at this geographic level, is unique.

The choice of a stochastic modeling approach [10] was based on the fact that it mimics the highly random initial phase of an epidemic better than does the traditional deterministic approach[11,12]. We first present the survey data used to estimate travel intensities between different municipalities in Sweden. We then introduce the simulation model for simulating the spread of the diseases and study the effect of travel restrictions. This introduction is to some extents a recapitulation of Hufnagel's model. Following this, we present the results of the simulations. We conclude our study with a discussion of the validity of the model and possible conclusions for future policy interventions.

## Methods

For this study, we used data from a random survey carried out by Statistics Sweden from 1999 to 2001, inclusive [13]. A total of 17000 individuals took part in the survey, constituting 71.9% of the selection. In all, 34816 distinct intermunicipal trips were reported. An intermunicipal journey was defined as a trip between two points where the individual lives, works, or conducts an errand. In other words, we treated a journey between home and work as several trips if the traveler made stops on the way for errands, provided that a municipal border was crossed between each stop. The data were weighted to correspond to 1 day and to the entire population for ages 6 to 84 years. For a more detailed description of the travel data, see Appendix A in the supplementary material.

As it turned out, roughly 1% of the data was significantly erroneous and was consequently removed*. From the remaining set, we estimated a travel intensity matrix with each element corresponding to the one-way travel intensity between two municipalities. The number of non-zero elements was 11611 (to be compared with the size of the matrix: 83521). The matrix elements stood in direct correspondence with the underlying data, weighted for time and population. Even though the matrix gives a good picture of the traveling pattern in Sweden, we must treat any travel intensity between two specific communities with care. This is especially true for small communities with only a single or very few journeys made between them.

**Figure 1:**
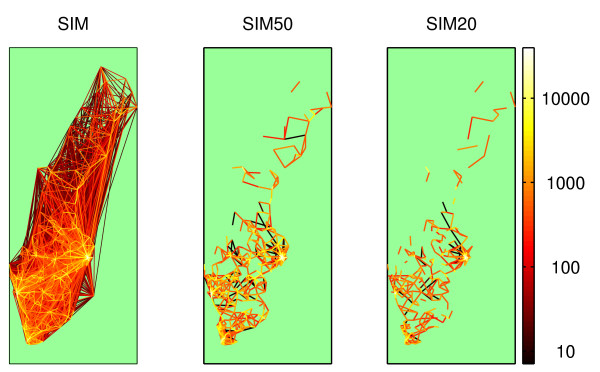
**The intermunicipal travel network**. The intermunicipal travel network with travel intensities indicated by color lines. The scale is logarithmic in trips per day. SIM shows the complete dataset. In SIM50 and SIM20, all journeys > 50 km and 20 km, respectively, have been removed. The lines are drawn between the population centers of each municipality, so in many cases the trips are shorter than the lines representing them.

A total of nine scenarios, with 1000 realizations each, was simulated to study the effects of three levels of travel restrictions as a control measure, for three different levels of the global intercommunity infectiousness parameter, *γ*, which was used to calibrate the model in the study of Hufnagel et al. Sixty days was chosen as the simulation period, as this gives sufficient time for a possible extinction to occur and for all stochasticity to play out its part in all but the smallest and most distant municipalities. Each scenario started with a single infectious individual in Stockholm, and treated the country as isolated from influx of disease. The traveling restrictions were divided into three levels. In the first level, we used the complete intensity matrix. In the following two, we removed data corresponding to journeys >50 km and journeys >20 km, respectively. The simulations were designated SIM, SIM50, and SIM20, respectively. In Figure 1, the datasets are displayed as geographical plots.

We also considered the case if the travel restrictions were not obeyed wholly by the public. Perhaps 5% might not heed the restrictions, resulting in a small but non-zero intensity for trips longer than the set restrictions. Full 1000-run simulations were made at varying levels of distance restrictions and compliance, resulting in a mesh surface of the incidence.

We used a simplified version of the model suggested by Hufnagel et al [8], and thus the following is as much a description of Hufnagel's model as our own. The individuals in both models can be in four different states:

▪ S: susceptible

▪ L: latent, meaning infected but not infectious

▪ I: infectious

▪ R: recovered and/or immune.

The rate at which individuals move from one category to the next is governed by the intensity parameters: *α *= 0.55^†^; *β *= 0.21, which is the inverse infectious time and *ν *= 0.19, the inverse latency time[14,15]. Individuals become infected at a rate proportional to *α *and the number of infected (force of infection). They subsequently become infectious at rate *β*, contributing to the force of infection, and recover at rate *ν*. Thus far, this is a description of a regular random-mixing epidemic model. We now assume a traveling component contributing to the force of infection, a term for each of the connected municipalities proportional to the number of infectious there.

As the process is assumed to be Markovian, as in Hufnagel's model, the time between two events, *Δt*, is random, taken from an exponential distribution,

Δ*t *∈ Exp(1/*Q*)     (1)

where Q is the total intensity, the sum of all independent transmission rates:

QiL=αIiSiNi+∑j≠iγj,iIjSiNi,QiI=νLi,QiR=βIi.Q=∑iQiL+∑iQiI+∑iQiR.     (2)
 MathType@MTEF@5@5@+=feaafiart1ev1aaatCvAUfKttLearuWrP9MDH5MBPbIqV92AaeXatLxBI9gBaebbnrfifHhDYfgasaacH8akY=wiFfYdH8Gipec8Eeeu0xXdbba9frFj0=OqFfea0dXdd9vqai=hGuQ8kuc9pgc9s8qqaq=dirpe0xb9q8qiLsFr0=vr0=vr0dc8meaabaqaciaacaGaaeqabaqabeGadaaakeaafaqabeabbaaaaeaacqWGrbqudaqhaaWcbaGaemyAaKgabaGaemitaWeaaOGaeyypa0dcciGae8xSdeMaemysaK0aaSbaaSqaaiabdMgaPbqabaGcdaWcaaqaaiabdofatnaaBaaaleaacqWGPbqAaeqaaaGcbaGaemOta40aaSbaaSqaaiabdMgaPbqabaaaaOGaey4kaSYaaabuaeaacqWFZoWzdaWgaaWcbaGaemOAaOMaeiilaWIaemyAaKgabeaaaeaacqWGQbGAcqGHGjsUcqWGPbqAaeqaniabggHiLdGccqWGjbqsdaWgaaWcbaGaemOAaOgabeaakmaalaaabaGaem4uam1aaSbaaSqaaiabdMgaPbqabaaakeaacqWGobGtdaWgaaWcbaGaemyAaKgabeaaaaGccqGGSaalaeaacqWGrbqudaqhaaWcbaGaemyAaKgabaGaemysaKeaaOGaeyypa0Jae8xVd4MaemitaW0aaSbaaSqaaiabdMgaPbqabaGccqGGSaalaeaacqWGrbqudaqhaaWcbaGaemyAaKgabaGaemOuaifaaOGaeyypa0Jae8NSdiMaemysaK0aaSbaaSqaaiabdMgaPbqabaGccqGGUaGlaeaacqWGrbqucqGH9aqpdaaeqbqaaiabdgfarnaaDaaaleaacqWGPbqAaeaacqWGmbataaaabaGaemyAaKgabeqdcqGHris5aOGaey4kaSYaaabuaeaacqWGrbqudaqhaaWcbaGaemyAaKgabaGaemysaKeaaaqaaiabdMgaPbqab0GaeyyeIuoakiabgUcaRmaaqafabaGaemyuae1aa0baaSqaaiabdMgaPbqaaiabdkfasbaaaeaacqWGPbqAaeqaniabggHiLdGccqGGUaGlaaGaaCzcaiaaxMaadaqadaqaaiabikdaYaGaayjkaiaawMcaaaaa@8446@

These are the equations that govern the simulations and give us the continuous time setting. The component *γ*_*j*,*i *_in (2) (note the reversed indexes) is the intermunicipal infectiousness corresponding to the one-way route *j *to *i*. If, ωj,i=Mj,i/∑iMj,i
 MathType@MTEF@5@5@+=feaafiart1ev1aaatCvAUfKttLearuWrP9MDH5MBPbIqV92AaeXatLxBI9gBaebbnrfifHhDYfgasaacH8akY=wiFfYdH8Gipec8Eeeu0xXdbba9frFj0=OqFfea0dXdd9vqai=hGuQ8kuc9pgc9s8qqaq=dirpe0xb9q8qiLsFr0=vr0=vr0dc8meaabaqaciaacaGaaeqabaqabeGadaaakeaaiiGacqWFjpWDdaWgaaWcbaGaemOAaOMaeiilaWIaemyAaKgabeaakiabg2da9maalyaabaGaemyta00aaSbaaSqaaiabdQgaQjabcYcaSiabdMgaPbqabaaakeaadaaeqbqaaiabd2eannaaBaaaleaacqWGQbGAcqGGSaalcqWGPbqAaeqaaaqaaiabdMgaPbqab0GaeyyeIuoaaaaaaa@40B5@ where *M*_*j*,*i *_is the travel intensity (i.e. *ωj, i *is the probability that a traveler in *j *will choose the route *j *to *i*, then *γ*_*j*,*i *_= *γω*_*j*,*i*_. In cases where restrictions are active, this expression is further scaled row-wise to match the smaller mass of the matrix. *γ *is the global intermunicipal infectiousness parameter mentioned above. We use the approximation given by Hufnagel et al based on data from the actual outbreak, *γ *= 0.27. The parameter *γ *is influenced by the total travel intensity, the medium of travel and as we have seen, the propensity for travel in different communities.

We would have liked to calibrate our model in a similar way, but as we have no outbreak data for Sweden, we needed to see whether changes in *γ *would drastically alter our conclusions. To get an idea of its effect, we compared Hufnagel's estimate of *γ *with other possible values. As *γ *is an infectiousness parameter playing a similar role in the governing equations above as *α*, we argue that *α *= 0.55 is an upper bound for *γ*. The force of infection between two municipalities with equal population and number of infectious is unlikely to be higher than from within those municipalities. To find an appropriate lower bound we extrapolated proportionately from these, producing 0.13.

Although this is not mentioned in the original work by Hufnagel et al, the expression above means that everybody, regardless of where they live, is equally prone to travel outside their home, the uptake area of the airport or, in our case, the municipality. This is a heavy assumption indeed, as it depends on the function of the municipality varying. The municipality may be a suburb or self-sufficient community, just as airports may be transit hubs or terminals. One of the strengths of Hufnagel's model is that it seems to be forgiving towards many simplifications, this one included, with the correct choice of *γ*. We investigated corrections for this assumption, such as row-wise scaling according to the known probability for travel, but found little effect on absolute incidence and none on the qualitative conclusions of the current study. As such, we were reluctant to stray from Hufnagel's model.

After the initial conditions were set up, including a single infected person in Stockholm, the simulation ran as follows. First, we moved forward in time with a random step *Δt *given by (1). We then selected the event that would occur with a probability proportional to the corresponding intensity. All intensities were updated according to the new state, and the process was repeated until the disease died out or the simulation period, 60 days, was passed.

## Results

**Figure 2:**
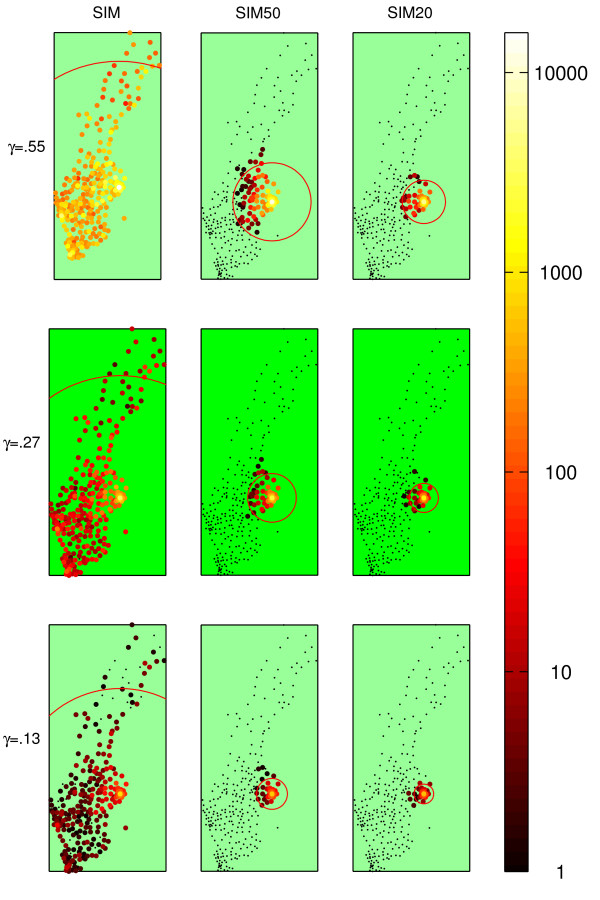
**Epidemic spread for different restrictions and values of *γ***. Geographical plot of the municipalities, logarithmically color-coded according to the mean incidence after 60 days. SIM depicts the complete data set. In SIM50 and SIM20, all journeys > 50 km and 20 km, respectively, have been removed. The red circle signifies the mean extent of the epidemic from Stockholm.

The results for all nine scenarios were plotted geographically and color-coded according to the mean incidence (Figure 2).

A scenario with no restrictions resulted in an outbreak in which a majority of the municipalities became affected regardless of *γ*. Only the incidence differed. A ban on journeys >50 km stifled the dynamics of the outbreak. For the two lower values of *γ*, we see that the disease remained in the Stockholm area after 60 days, and for the higher value of *γ*, the disease did not manage to spread far from the densely populated areas around the largest Swedish cities. Prohibiting journeys >20 km would result in an even slower spread with a small number of afflicted municipalities, mainly localized around Stockholm. What is more, the total incidence after 60 days as well as the incidence in each municipality dropped as we imposed the restrictions.

Table 1 compares the country's total incidence in the three simulations for which Hufnagel's estimate of *γ *was used. Table 2 presents the incidence broken down into a few selected municipalities.

**Table 1:** Main results

	SIM			SIM50			SIM20		
Results	Mean	95% SI		Mean	95% SI		Mean	95% SI	

Total number of infected	320 555	301 587	339 243	154 517	145 664	163 678	64 307	60 326	68 293
Percentage of population	3.6	3.4	3.8	1.7	1.6	1.8	0.72	0.67	0.76
Intermunicipal infections (n)	0.3	0.3	0.3	0.3	0.3	0.3	0.2	0.2	0.2
Incidence after 60 days (n)	77 184	72 760	81 784	37 065	34 941	39 321	15 240	14 307	16 190
Percentage of population	0.9	0.8	0.9	0.4	0.4	0.4	0.17	0,16	0.18
Afflicted municipalities (n)	262.1	258.5	265.4	47.2	46.6	47.8	34.0	33.6	34.5
Mean incidence in municipalities (n)	267.1	251.4	283.1	128.3	120.5	136.0	52.7	49.5	56.1
Mean influence distance (km)	1 222	-		245.1	-		153.8	-	
Travel intensity matrix	Value			Value			Value		
Total travel intensity (millions/day)	4.2	-		2.9	-		1.5	-	
Intermunicipal one-way routes (n)	11 611	-		1 386	-		797	-	
Summary	Value			Value			Value		
Extinction runs (n)	262	-		268	-		305	-	
Mean time for extinction (days)	3.48	2.84	4.14	3.48	2.78	4.25	3.61	2.85	4.46
Mean number of afflicted municipalities before extinction (n)	1.33	1.26	1.41	1.29	1.21	1.36	1.27	1.22	1.34
Total number of realizations	1 000	-		1 000	-		1 000	-	

The table shows the main results along with miscellaneous information about the simulation.

Figures refer to simulated values at the end of the run, 60 days. The mean, where applicable, was taken over the set of runs that ran their course through the full 60 days.

The extinction runs hence did not affect the means but their numbers are of course interesting in their own right.

The 95% simulation intervals (SI) were calculated by bootstrapping 10 000 samples.

By incidence, we mean the number of infectious people.

Intermunicipal infections is the percentage of the total number of infected that caught the disease via intermunicipal infection.

There are 289 municipalities in Sweden and the population is approximately 8.9 million.

**Table 2:** Municipalities of key interest

	SIM			SIM50			SIM20		
Municipality	Mean	95% SI		Mean	95% SI		Mean	95% SI	

Stockholm	18 563	17470	19645	13 231	12437	14066	6029	5653	6412
Göteborg	730.7	654.4	813.9	-	-	-	-	-	-
Malmö	338.6	295.4	390.2	-	-	-	-	-	-
Huddinge	3473	3277	3668	2607	2453	2761	1218	1136	1298
Upplands-Bro	573.7	537.0	610.7	362.2	337.7	388.1	84.1	76.2	92.4
Norrtälje	939.1	882.0	998.2	214.6	197.9	232.3	37.4	33.5	41.8
Södertälje	1133	1060	1205	638.2	593.5	685.1	60.7	51.4	72.3
Västerås	864.4	798.9	934.1	27.0	23.1	31.3	2.9	1.9	4.0
Eskilstuna	692.4	639.8	748.8	60.7	53.4	68.9	26.0	22.1	30.5
Umeå	118.2	98.1	144.6	-	-	-	-	-	-
Luleå	237.4	201.9	278.4	-	-	-	-	-	-
Örebro	557.0	507.7	611.1	0.3	0.1	0.4	-	-	-
Jönköping	227.6	206.4	250.9	-	-	-	-	-	-
Linköping	528.5	479.5	582.6	1.7	1.3	2.3	-	-	-
Helsingborg	143.0	128.9	158.3	-	-	-	-	-	-
Borås	140.3	127.6	154.5	-	-	-	-	-	-
Gävle	559.2	517.5	601.1	21.9	18.7	25.5	1.8	1.3	2.4
Ljungby	29.7	26.7	33.0	-	-	-	-	-	-
Hofors	72.9	66.8	79.2	2.9	2.1	3.9	-	-	-
Örkelljunga	4.9	3,8	5,0	-	-	-	-	-	-

A selection of municipalities with the mean incidence and bootstrapped 95 % simulation intervals with extinction runs filtered out. This set and its ordering is the same for the individual rows in the table, which explains the zero-valued lower interval bounds and other discrepancies.

After Stockholm, Göteborg and Malmö are the largest cities in Sweden. The single most traveled route is that between Stockholm and neighboring Huddinge, traveled by approximately 37 000 people daily, each way. The decline in incidence closely follows that in Stockholm.

Upplands-Bro is representative of an outer suburb to Stockholm. Södertälje and Norrtälje are nearby towns but are not considered suburbs. Västerås and Eskilstuna are more distant, but have a fair number of commuters. Örebro through Luleå are larger towns at some distance from Stockholm with no notable commuter traffic. Finally, the last four are small towns in southern Sweden.

SI, simulation intervals

The reason for the decrease in incidence is of course the limited transmission paths available to the disease. The disease, after having spread from one municipality to another will constantly be transmitted back into the originating municipality, provided that there is a flow of travelers in the opposite direction in the travel intensity matrix. Travel restrictions limit both spread to other municipalities and reintroduction. For comparison, if traffic is removed altogether, the mean incidence in Stockholm will be 917.

The process outlined above is also responsible for the decreased number of extinction runs. For a regular continuous time branching process where the number new cases is completely independent between individuals, one would expect the probability of extinction to be I/R_0 _= 37%. and this was confirmed in the course of testing our model.

It is also clear how travel restrictions confer increasing protection on cities that are further from the capital, the focal point of the infection. The major cities of Göteborg (Gothenburg) and Malmö would be protected even though traffic into these cities is heavy. In fact, the farthest the disease woudl ever make it in SIM50 is Ljungby, 1471 km from Stockholm and still some 200 km from Malmö. For SIM20, the farthest city is Uddevalla, 441 km away and a suburb of Göteborg. The mean reach of the epidemic in those cases is only 276 and 34 km, respectively.

**Figure 3:**
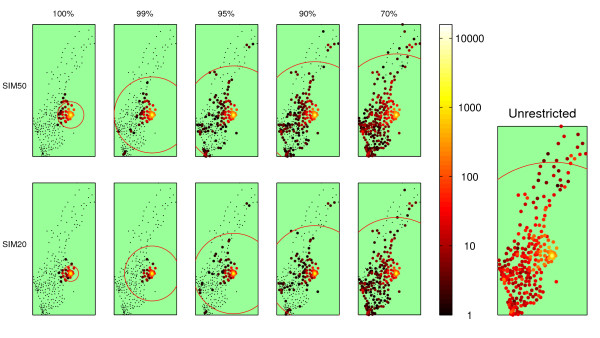
**Epidemic spread for different restrictions and compliance**. Geographical distribution of the incidence after 60 days shown for SIM50 and SIM20 for different levels of compliance. The left plot shows the unrestricted case with Hufnagels original *γ *galue for comparison. This plot reflects the same data as that on the middle row, right column of Figure 2 but with scale to match the current figure.

**Figure 4:**
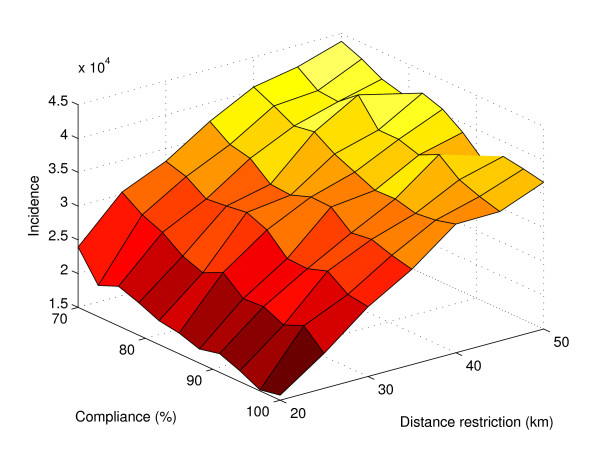
**Total incidence for varying compliance and restrictions**. A surface plot showing incidence after 60 days with the parameters of compliance and distance restrictions on the data axes. 1000 realizations were made for each point. The surface has its highest values at high set distance limit and low compliance. Its low values are found at opposite corner.

An objection to the applicability of this model is that in all probability, complete enforcement of the restrictions may not be achievable or even desirable, as in the case of high-priority professionals with crucial functions in society during a crisis situation. Incidence does indeed climb the more restrictions are ignored, but not to such an extent as to render the travel restrictions dubious as a means of disease control (Figure 3). A plot with unrestricted travel, duplicated from Figure 2, is given for comparison. Figure 4 shows a finer spaced mesh of incidence versus restriction distance and compliance. Bear in mind that there was no attempt to correlate the randomness between the simulation sets. Therefore, the random numbers used in each were completely independent, giving rise to considerable simulation noise. Even though the landscape is rough, the trend in both dimensions was clearly visible. Looser travel restrictions and lower compliance means higher incidence.

## Discussion

Our results show clearly that traveling restrictions would have a significant beneficial effect, reducing both the geographical spread and the total and local incidence. This holds true for all three levels of intercommunity infectiousness, *γ*, simulated, *γ *is influenced by many factors, most notably by total travel intensity, but also by the medium of travel, the behavior of the traveler, the model of dispersal by travel and the infectiousness of the disease. Hufnagel et al calibrated *γ *using data from the actual outbreak. As mentioned, no attempt was made on our part to find the "true" value of *γ *in the new settings, as no such outbreak data are available for Sweden. This would be considered a flaw for a quantitative study on a SARS outbreak in Sweden. By simulating for different values of the parameter, however, we can be confident in the qualitative conclusion, namely, that the same general behavior can be expected in the unrestricted scenario and in response to the control measures, regardless of *γ*.

The same reasoning supports generalization of the results to other countries or regions. The survey travel data give a fairly accurate picture of travel patterns in Sweden and mirror many western countries. Some countries, e.g. the USA, are more dependent on motor vehicles for commuter traffic, and infectiousness would therefore be anticipated to be lower. Such effects are, again, included in calibration of *γ*. Keep in mind that the used value of *γ *was taken from a model including only global air traffic. Figure 2 shows that travel restrictions have a positive effect for *γ *in the proposed range.

In light of the fact that intermunicipal travel heavily influences incidence even at a local level, we may justifiably be concerned about boundary conditions. We treated Sweden as an isolated country, but quite obviously, the incidence will be underestimated for areas with frequent traffic across the borders. This includes in particular the Öresund region around Malmö, and to a lesser extent, international airports and the small towns bordering Norway and Finland.

We would like to point out that, as in most epidemiologic simulations, individuals are not explicitly represented in the model. This is also true for individuals who are traveling. In reality, people who travel run an increased risk of contracting the disease. This is correctly modeled, as individuals who are traveling are included in the travel influx into the municipalities. The influx in turn affects the probability of additional infections at any given time. Of course, it is highly probable that this would be the travelers themselves, as, almost without exception, they return to the origin of their journey.

Even though there is presently no treatment or vaccine for SARS, results show that limited quarantine as suggested here drastically decreases the risk of transmission, and this may well turn out to be the most expedient form of intervention. In many countries, Sweden included, limiting freedom of travel is unconstitutional and must take the form of general recommendations. Additionally, certain professions of crucial importance to society during a crisis situation must be exempt from travel restrictions. The study shows that even if a substantial fraction of the population breaks the restrictions, this strategy is still viable. For other types of disease for which preventive treatment (pandemic flu) or vaccine (smallpox) are available, our results show that long-distance travelers are an important group for targeted control measures.

It is worth noting on the travel intensity matrix, where the elements directly reflect the underlying survey data, that there are several proposed alternatives, using smoothing techniques or data-generating simulation. Completing the matrix in such a way would correctly introduce many connections that are missing from our data, but a substantial number would be falsely represented, and could endanger the validity of the model due to unforeseen stochastic mechanisms. The methods all have inherent imprecisions and flaws, which unfortunately in this context would be difficult to estimate. The choice of one in preference over another would certainly be contended. Our scheme of direct extrapolation from the raw data is certainly no better but does have the benefit of transparency and reasonable control over errors. As is explained in further detail in the appendix, this means that certain connections between municipalities that are used in reality, however infrequently, are missing, while on the other hand some will be heavily overestimated. This is especially true for certain unusual municipalities. The routes between the more populated communities and other heavy connections are much better estimated, as crude statistical analysis will indicate. Also close to the true value is the travel intensity as a whole, as well as the summed influx and outflux of any municipality.

## Conclusion

Our methods show that restricting travel between municipalities in such a way that travel above a certain distance is banned, would indeed have a beneficial effect on the speed of transmission of a highly contagious disease, geographically and in absolute numbers. This conclusion is true for a range of plausible values of the intermunicipal infectiousness. Even in scenarios of compliance as low as 70%, travel restrictions are effective. Thus, the effectiveness of travel restrictions as a means of mitigating a future epidemic is supported. The model and results are robust and there is no reason to believe that the results are not generally applicable to any country or region.

## Competing interests

The author(s) have received financial support from the organizations mentioned in acknowledgements.

## Authors' contributions

MC performed all coding and simulations, carried out analyses and is the main author of the manuscript. FL conceived the project and design and initiated the work. He participated in analyses and drafting of the paper. FL approved the final version of the paper.

## Notes

* The erroneous records were long-distance journeys, mostly between individual communities in an unreasonably short time. Had they not been removed, their influence would have been significant, accelerating the spread across the country. The correct data were irretrievable but the effect of their absence was deemed within the margin of error for long-distance journeys.

^† ^Some authors refer to this as the "attack rate" although this is not the commonest definition.

## Supplementary material

Appendix A

This appendix describes the travel survey data from Statistics Sweden in greater detail.

## Pre-publication history

The pre-publication history for this paper can be accessed here:



## Supplementary Material

Additional File 1 **Sample animations**. The animations in wmf format provided with this manuscript show different single realizations or possible scenario of an unrestricted epidemic where one initial infected person is located in Stockholm. They are provided for interest only and should not be seen as contributing results, which require the combined analysis of many more realizations. The first file, Stockholm.wmf, shows a common scenario of spread throughout the country from Stockholm. Click here for file

Additional File 2 **Sample animations**. The animations in wmf format provided with this manuscript show different single realizations or possible scenario of an unrestricted epidemic where one initial infected person is located in Stockholm. They are provided for interest only and should not be seen as contributing results, which require the combined analysis of many more realizations. Uppsala.wmf clearly demonstrates the benefits of stochastic modeling in that the epicenter is spontaneously translocated to Uppsala, north of Stockholm, and the epidemic is hence delayed. This is quite a plausible event that would not be captured in deterministic models. Click here for file

Additional File 3 **Sample animations**. The animations in wmf format provided with this manuscript show different single realizations or possible scenario of an unrestricted epidemic where one initial infected person is located in Stockholm. They are provided for interest only and should not be seen as contributing results, which require the combined analysis of many more realizations. Dies_out.wmf shows the common event of the epidemic not catching on but rather dying out within a few days. All these realizations have been aborted after 60 days. Click here for file

Additional File 4 **Sample animations**. The animations in wmf format provided with this manuscript show different single realizations or possible scenario of an unrestricted epidemic where one initial infected person is located in Stockholm. They are provided for interest only and should not be seen as contributing results, which require the combined analysis of many more realizations. Biggy.wmf is similar to the first but was not aborted until the epidemic has peaked and died out due to depletion of susceptible individuals. Click here for file
